# How Can We Best Assess Spatial Skills? Practical and Conceptual Challenges

**DOI:** 10.3390/jintelligence12010008

**Published:** 2024-01-16

**Authors:** David H. Uttal, Kiley McKee, Nina Simms, Mary Hegarty, Nora S. Newcombe

**Affiliations:** 1 Department of Psychology, Northwestern University, Evanston, IL 60208, USA; 2 Spatial Intelligence and Learning Center, Northwestern University, Evanston, IL 60208, USA; 3 Department of Psychological & Brain Sciences, University of California, Santa Barbara, CA 93106, USA; 4 Department of Psychology and Neuroscience, Temple University, Philadelphia, PA 19122, USA

**Keywords:** spatial cognition, assessment and measurement, psychometrics

## Abstract

Spatial thinking skills are associated with performance, persistence, and achievement in science, technology, engineering, and mathematics (STEM) school subjects. Because STEM knowledge and skills are integral to developing a well-trained workforce within and beyond STEM, spatial skills have become a major focus of cognitive, developmental, and educational research. However, these efforts are greatly hampered by the current lack of access to reliable, valid, and well-normed spatial tests. Although there are hundreds of spatial tests, they are often hard to access and use, and information about their psychometric properties is frequently lacking. Additional problems include (1) substantial disagreement about what different spatial tests measure—even two tests with similar names may measure very different constructs; (2) the inability to measure some STEM-relevant spatial skills by any existing tests; and (3) many tests only being available for specific age groups. The first part of this report delineates these problems, as documented in a series of structured and open-ended interviews and surveys with colleagues. The second part outlines a roadmap for addressing the problems. We present possibilities for developing shared testing systems that would allow researchers to test many participants through the internet. We discuss technological innovations, such as virtual reality, which could facilitate the testing of navigation and other spatial skills. Developing a bank of testing resources will empower researchers and educators to explore and support spatial thinking in their disciplines, as well as drive the development of a comprehensive and coherent theoretical understanding of spatial thinking.

## 1. Introduction

Spatial thinking is important in many kinds of human cognition and learning. It involves a variety of skills, including encoding and mentally manipulating the shapes and locations of objects, their relations to each other, the paths they take as they move, and the paths we take as we move in, around, and through larger objects (e.g., buildings) and over terrain. In addition, it involves symbolic systems, including language, maps, and diagrams, that encode spatial information for reflection and communication with others and that can reveal new insights.

In the past 15 years, research on the role of spatial thinking in learning and practice in STEM fields has experienced transformative growth. Researchers in a variety of STEM disciplines, including geology, physics, chemistry, mathematics, and engineering, have highlighted the role of spatial thinking in their fields. Longitudinal studies of spatial skills show that they correlate with participation and success in STEM careers, even after controlling for differences in verbal and mathematical intelligence, both in young adulthood (Shea et al. 2001; Wai et al. 2009) and in childhood (Frick 2018; Verdine et al. 2017). Spatial skills uniquely account for significant variance in scientific creativity, even after accounting for verbal and mathematical skills (Kell et al. 2013; Atit et al. 2022). Sex differences in spatial skills assessed in elementary school are related to sex differences in STEM participation at university (Tian et al. 2022).

Moreover, spatial skills can be improved through training, education, and life experience (Uttal et al. 2013). This improvement is evident in both adults and children and both men and women (Lowrie et al. 2017, 2018, 2019, 2021; Mix and Cheng 2012; Uttal et al. 2013). Findings regarding malleability are particularly interesting because they suggest the potential to use spatial thinking interventions and training to improve learning in STEM domains (Hawes et al. 2022) and help improve equity and diversity in STEM, including addressing the gender gap (Tian et al. 2022). The training of spatial thinking skills can transfer to an array of STEM-relevant skills and endure over time (Hawes et al. 2022; Sorby et al. 2018; Uttal et al. 2013). Such findings have generated substantial excitement about developing spatial pedagogical techniques to facilitate STEM learning, and several research programs are underway to evaluate these programs. The field is ready to move to a better, more precise, and more actionable understanding of spatial thinking (Mix 2019). To do so, researchers and educators must be empowered to explore and support spatial thinking in their disciplines, which, in turn, requires a clear understanding of what comprises spatial thinking and effective ways of assessing it.

Historically, most spatial tests were developed to address a specific need. For example, during World War II, millions of new volunteers and conscripts had to be placed into jobs in the military, and attempts were made to use spatial skills tests (among many others) to facilitate these placements (Hegarty and Waller 2005). Recruits with high spatial or mechanical skills, for example, might have been assigned to jobs such as engine maintenance and repair or navigation. As important as these tests were, the focus on addressing specific problems usually led to a test that was not motivated by a coherent theory regarding (a) what spatial thinking is or (b) when and how it should be measured. The result is a motley mix of many spatial tests and a hit-or-miss approach to selecting them. Furthermore, many of these tests are difficult to access or have incomplete psychometric information, especially for groups other than typically developing young adults. These issues pose significant barriers to further developing our understanding of how spatial thinking functions, how it can be improved, and how it can support success in STEM and other domains. In short, limitations in spatial testing restrict our ability to realize the full potential of talent in STEM.

In this roadmap report, we outline problems in spatial research using such tests based on a combination of our own observations and the results of our interviews and surveys with colleagues and STEM experts. We also discuss origins and solutions. The practical problems with testing are reciprocally related to the challenge of characterizing the nature of spatial skills, and addressing one problem will synergize with finding solutions to others. Our aim is to articulate plans for how the field of spatial thinking can achieve its transformative potential.

Our vision of what our proposed work could accomplish is closely aligned with the strategic goals outlined by the National Science Foundation (NSF) (NSF 2022-2026 Strategic Plan): to empower STEM talent to fully participate in science and engineering; to discover new knowledge about how people think spatially and how spatial thinking shapes our experiences; and to make an impact by translating this knowledge into solutions to benefit society. Here, we discuss how improvements and increased access to spatial tests could realize these goals—for spatial thinking and for STEM learning more generally.

## 2. Limitations of Current Spatial Tests

Anyone conducting research on spatial thinking over the last three decades has likely received frequent questions from other researchers regarding which spatial test to use to assess a particular skill or where to find a specific test. These inquiries suggest that researchers are interested in using spatial tests but do not know which tests are appropriate or how to access them. Furthermore, many new tests have been developed and used by individual researchers, but often without adequate psychometric information, such as their reliability and validity, and typically without open access. Based on these anecdotal observations, we sought to get a better idea of the nature and dimensions of the problem by interviewing or surveying researchers who use spatial tests in their research. Specifically, we surveyed 48 individuals and interviewed 15 individuals who were either notified about the opportunity from the Spatial Intelligence and Learning Center (SILC) listserv, which includes individuals interested in spatial thinking and STEM education, or the Cognitive Development Society (CDS) listserv, which includes members who are interested in cognitive development across the lifespan. We stress that these surveys and interviews were not designed to be a systematic assessment of a representative sample of researchers. Rather, our goal was to confirm our own observations and anecdotes by reaching out to a group of other researchers who frequently use, or want to use, spatial tests in their own work. We asked researchers about the spatial tests that researchers use, the purpose they are used for, and their knowledge and experiences with using the tests (see Appendix A). For each test, they were asked what the test measured (e.g., perspective taking), the age group the test was appropriate for, whether there was a manual or set of detailed instructions for administration available, and whether the test had been normed and/or validated. For their most used tests, the researchers were also asked several questions about how the test is administered, how they obtained access to the test, how they use the test in their research, the advantages and disadvantages of using the test, and any caveats about the test. Finally, the researchers were asked what factors had prevented them from using spatial tests in the past, and they were invited to share their thoughts about the state of spatial tests in the field.

We also held more open-ended focus groups with 15 researchers with interests in spatial thinking, in the context of focus groups. These individuals included six professors, two postdoctoral scholars, and seven graduate students, with research areas including STEM education (technology, geoscience, mathematics) and cognitive psychology. The participants were asked about the tests they were currently using, how they accessed the tests, and their purpose for using the tests. They were also asked about theories and taxonomies that informed their research and to suggest resources, including new tests and online repositories, that might facilitate their research.

The researchers’ responses were categorized into five major areas of concern: accessing tests, missing test information, tests not being available, tests being dated, and theoretical issues with tests. Once these areas were determined, more specific concerns within each category were identified. Additionally, the more concrete answers, such as specific test usage, were examined through the lens of the identified issues. Here, we detail these major problems with the current state of spatial assessments.

### 2.1. Lack of Access Is a Significant Barrier

The researchers’ answers revealed substantial difficulty in gaining access to existing tests.

#### 2.1.1. Researchers Do Not Know How to Find and Select Tests

Researchers reported that the tests they use were chosen largely based on availability and use by other researchers. The researchers learned about tests from reading the literature and received them from the researchers who developed the tests, other researchers who used the tests, or vendors. Several researchers noted that when they or students and colleagues were searching for tests, they would refer them to the resources page of Northwestern University’s Spatial Intelligence and Learning Center (SILC—formerly funded by NSF), which provides a list of spatial tests, tools, and software developed by SILC members and others (and, in some cases, also provides the tests). However, 69% of the respondents did not know how to obtain tests and 38% reported not knowing where to obtain permission to use tests (i.e., who held the copyright).

#### 2.1.2. Spatial Tests Are Often Expensive

Several participants also said that the costs of proprietary tests are prohibitively expensive. In our interviews, participants noted that in some cases, they might have been willing to buy tests if they could have seen sample versions of these tests in advance, but test companies would not provide samples.

#### 2.1.3. Other Researchers Do Not Always Share Their Tests

Although researchers reported that the tests they use were often received from other researchers, several participants also described difficulty procuring tests directly from the researchers who created them. One respondent noted that they generally assumed that accessing measures encountered in the literature would not be possible unless the test was already being shared somewhere—attempts to acquire tests directly from the authors frequently resulted in no reply or replies stating that the original test and materials could not be located to share. Another respondent observed that there is fragmentation between labs and reliance on separate “testing ecosystems” that use lab-grown tests targeting a small number of hypotheses. Even gaining access to tests from testing companies is sometimes difficult—one focus group said they enquired about purchasing tests from the testing companies and received no response.

### 2.2. Critically Important Information about Tests Is Lacking or Very Difficult to Find

Once a test has been secured, researchers often lack basic psychometric information.

#### 2.2.1. Many of the Tests Have Not Been Validated or Normed

In our structured interviews, the 48 researchers we surveyed listed 170 spatial tests (with many tests listed by multiple participants). Only 63% of these tests were reported as validated and 38% of the tests were reported as normed. In addition, for 38% of the tests, respondents reported not being sure whether the test was validated or normed—that is, validation and norming information was not sought or could not be found for over a third of the tests used by this sample of researchers. However, researchers often listed reliability and validity as important considerations in choosing tests. Indeed, only just under half of the respondents cited a lack of information about test properties as a reason for not using a spatial test. Moreover, relatively little work is being performed to vet spatial tests.

#### 2.2.2. Administration and Scoring Are Often Not Documented or Standardized

Having clear and comprehensive instructions (and codes) for administering and scoring tests is obviously necessary to properly use spatial tests. Our interviewees also noted that standardized procedures are critical for interpreting results. In line with this, most respondents reported that while the tests they were using did have manuals or instruction booklets, this documentation was often found to be lacking or unclear in many ways. About half of the participants reported a lack of standardization information, including standardization for specific populations, as a reason they did not use a particular test. Some noted confusion over what methods should be used or concerns about how different methods may affect the interpretation of results. Information about “lab-grown” tests that are developed by individual researchers or labs for their own purposes is typically limited to what has been published in empirical reports, which rarely include sufficient detail on items, administration, or scoring procedures. Information regarding administration and scoring is usually much better for propriety tests, resulting in researchers having to choose between reliable administration and affordability.

### 2.3. Some Critically Important Needs Are Not Met by Current Tests

Our respondents also noted that they wanted to assess constructs for which there did not seem to be assessments, despite the multiplicity of spatial tests.

#### 2.3.1. Researchers Need Tests for a Wider Range of Populations, Contexts, and Purposes

Participants commented on the need for tests appropriate for a large age range—in particular, researchers expressed a need for tests for young children, but also for tests that can be used across the lifespan. Tests that could be used across cultures and languages were also an issue. Researchers had concerns about cross-cultural reliability and translating task instructions—one suggestion was to use graphical, rather than verbal, instructions to make it easier to use tests across different cultural and language groups.

Many researchers also wanted tests that were appropriate for use in the classroom. One interviewee remarked that they wanted to use spatial tests formatively, but such use requires tests that can be scored easily and quickly—ideally, automatically—to provide timely feedback, especially for large groups of students (e.g., undergraduate lecture courses). The need for spatial tests that were quick to administer was also often mentioned in responses during the structured and open-ended interviews. Some focus groups stated that they would like an efficient test that measures several aspects of spatial thinking but does not take too long to administer. They would like this test to be diagnostic so that it gives the test taker feedback on what aspects of spatial thinking they are good or bad at, and which enables the researcher to diagnose the test taker’s strategies. The respondents also called for parallel forms of tests, adaptive tests that adapt to the age and/or ability of the test taker, and gamified tests (noting the current bias against aptitude testing).

#### 2.3.2. Researchers Often Must Create Their Own (Versions of) Tests

Our respondents reported making many changes to tests to make them appropriate for the questions or ages they were studying. These changes included the following: adapting tests designed for adults to be used with young children; adapting tests designed for individual administration to be used in group settings (e.g., in classrooms); creating abridged versions of tests; creating computerized or web versions of paper/pencil tests; creating new tests to more adequately capture the skills used in a particular discipline; and re-making tests found in the literature that could not be obtained. However, as one researcher noted, those adapting or creating new tests may not have the expertise in psychometrics required to create reliable, valid measures. Additionally, researchers may not be interested in investing the significant time and effort that can be needed to discover and refine important test properties.

### 2.4. Existing Tests Are Dated

#### 2.4.1. Tests Neglect Modern Advances in Spatial Research

Many (or even most) assessments of spatial thinking in STEM education focus on 3D mental rotation, using either the Vandenberg and Kuse (1978) mental rotation test or the Purdue Spatial Visualization Test (Guay 1976). Our interviewees agreed that mental rotation tests were the most used spatial tests—most interviewees listed at least one test of mental rotation, excluding tests like Ramful et al.’s (2017) Spatial Reasoning Instrument that includes but does not exclusively target mental rotation. The predominance of mental rotation tests is highlighted by the fact that the next most common type of test, tests of perspective taking, was listed by only half as many interviewees. The popularity of mental rotation tests may be in part because they meet many of the criteria researchers have for selecting tests, including wide use by other researchers, short administration duration, and ease of administration.

However, there are serious doubts regarding whether these tests effectively encompass many of the skills that are needed for spatially intensive aspects of STEM learning. For example, research in the geosciences suggests that important spatial skills involve transformations, such as non-rigid shearing, bending, and folding, that are not assessed in traditional spatial tests, almost all of which emphasize the mental manipulation of solid structures that are transformed in standard Euclidean ways (e.g., rotation, scaling, translation, etc.). Moreover, men, on average, perform substantially better than women on the Vandenberg and Kuse mental rotation test, but men and women perform comparably on many other spatial tests (Voyer et al. 1995). The focus on mental rotation may lead researchers to focus on deficits instead of strengths and to emphasize an essentialist perspective on sex, gender, and gender differences (Bartlett and Camba 2023). Moreover, general reports of the psychometric characteristics of the tests, though available, have not been updated in decades and, hence, we may be making choices about which tests to use based on dated or incorrect information.

#### 2.4.2. Testing Methodology Is Not up to Date

Many respondents saw an urgent need for online tests. However, according to our interviews, only 32% of the most used tests can be administered via the internet, even though 58% were computer-based. Thus, even when tests could easily be administered online, they were not. Moreover, tests often used paper/pencil administration (58%), which limits the flexibility and scale of data collection. Recent advancements allow for more flexible and robust testing, including adaptive testing, algorithmic item generation, and gamification. Increased computing capability and access provide new possibilities for displaying dynamic and interactive stimuli. The need for even simple improvements, like re-drawing figures from classic tests that are unclear and of low fidelity, was noted by several interviewees.

#### 2.4.3. We Do Not Know Whether Current Tests Are Appropriate for Diverse Populations

Many spatial tests were developed on so-called “WEIRD” samples (white, educated, industrialized, rich, and democratic), that do not represent most of the people(s) of the world. Diversity may be particularly important in the spatial realm for at least two reasons. The first concerns the relation between the tests that are used and the appearance of gender differences. For example, sex differences have been found in tests including but not limited to mental rotation, navigation, and visual–spatial working memory (Nazareth et al. 2019b; Voyer et al. 1995, 2017). However, there is continuing debate regarding which tests consistently identify sex differences (Bartlett and Camba 2023; Hyde 2005), whether these differences are meaningful for STEM achievement, and whether the sex differences are decreasing. The second concerns the possibility that “traditionally” underprivileged groups may do particularly *well* on spatial measures. For example, Coutrot et al. (2022) have found that individuals from rural communities performed better on tests of navigation, and they may also perform better on other spatial tasks (e.g., Norman 1980; Wai and Lakin 2020). However, spatially talented students often receive less attention and are less engaged in school than students with different intellectual profiles. Identifying spatially talented students may therefore allow educators to provide more relevant and appropriate materials and curricula that these students may find more engaging (Wai and Lakin 2020). Likewise, individuals diagnosed with autism may do particularly well on spatial assessments (e.g., Kunda and Goel 2011). Some spatial tests do seem to be valid with non-WEIRD samples. For example, some spatial tests correlate with mobility among Twe and Himba people, suggesting that spatial testing could be further extended to more diverse samples (Vashro and Cashdan 2015).

### 2.5. Researchers Do Not Know Which Tests Are Appropriate for Which Questions

#### 2.5.1. Researchers Lack Clarity on Specific Tests and What They Measure

Researchers often ask about including spatial measures, but they are not sure which tests measure which constructs. There is no clear consensus within STEM fields as to what the different tests are intended to measure or how well they do so. For example, our respondents often listed tests without a specific test name—for example, “mental rotation” instead of “Purdue Spatial Visualization Test” or “Vandenberg and Kuse Mental Rotation Test”. This finding could indicate that researchers were unaware of different tests that measure similar constructs that would need to be differentiated or that they thought that differences between tests did not matter. In addition, the constructs measured by the same tests were sometimes described differently by different researchers. For example, the constructs measured by the Paper Folding Test were described both as “spatial visualization” and “transformational ability”; those measured by the WPPSI Block Design were described as “spatial visualization” and “pattern re-creation”.

Several interview participants also raised the question of different strategies used on spatial tests. One interviewee emphasized that spatial skills are not a “natural kind” (see also Newcombe 2018)—just because tasks have spatial properties does not mean they are solved the same way, with the same processes. Confusion about what spatial tests measure may be especially pronounced for STEM educators who are not experts in spatial thinking per se. Although the STEM educators we interviewed were broadly interested in understanding and fostering spatial thinking, they also relied on more expert colleagues and collaborators to identify and provide appropriate spatial tests. To make progress, we need clear and accessible definitions and examples of different spatial tests and how and when they have been used in studies of STEM achievement and attainment. As one participant put it, “I would like to know enough about spatial ability that we can liberate someone’s spatial ability to be useful to them. This is more important than figuring out the instruments”.

#### 2.5.2. We Do Not Know How Different Tests Relate to Each Other

It has been very difficult to determine how different spatial tests relate to each other (see Carroll 1993; Hegarty and Waller 2005; Linn and Petersen 1985; Newcombe and Shipley 2015; Uttal et al. 2013). What tests measure the same thing, and what tests are distinctly different? Such information is of critical importance in guiding questions about which tests should be used to measure different aspects of STEM learning and practice. Even tests with similar names often measure different constructs. For example, Brucato et al. (2023) recently studied four common measures of perspective taking and found that one of the tests measured a distinct skill compared to the other three.

Researchers want to understand how spatial skills are related to one another to understand underlying cognitive processes. Researchers also need this information to make practical decisions. One STEM educator we interviewed, for example, was focused on finding the most effective and efficient way to use tests to support students’ spatial skills in their domain. They wondered which skills must be measured (and supported) individually and which overlap so that testing (or training) one gives you some purchase on other related skills. They also wanted to know whether there are important differences between domain-general and domain-specific spatial tests.

#### 2.5.3. We Do Not Know What Tests Are Missing, That Is, What Tests Still Need to Be Developed

Despite the large number of existing tests, there are many important constructs for which there are not adequate measures or tests. For example, collaborative research between geoscientists and cognitive scientists has highlighted the importance of non-rigid transformations, but there are few if any good assessments of the relevant skills (e.g., Atit et al. 2013, 2020). In another example, researchers interested in technology education who participated in our focus group interviews expressed a need for a test of spatial communication—that is, the ability to externalize an internal spatial representation. Careful examination of the spatial demands of other STEM disciplines is likely to identify other aspects of spatial thinking that are not currently being measured by available spatial tests.

## 3. Conceptual Problems

A significant barrier to progress in research on spatial thinking and a root cause of many of the problems mentioned above has been the lack of a coherent theory or organizing model of spatial skills and what spatial tests assess. Despite more than a century of research, there is little consensus regarding what different spatial tests measure or how they relate to one another (Hegarty and Waller 2005). The lack of a coherent theory has resulted in, and from, a fragmented field, with different research groups, STEM disciplines, and even geographical regions pursuing work on spatial thinking with different sets of tools and assumptions. Consequently, test users often struggle to determine which spatial construct they should measure to address a given question and which test best measures this construct. This confusion is particularly strong for researchers, such as STEM scholars, who are not educated in cognitive psychology or psychometrics and may have little experience in choosing tests.

### 3.1. Lack of Consensus across Frameworks

Historically, approaches to classifying tests relied on exploratory factor analysis (e.g., Carroll 1993; Lohman 1988; McGee 1979; Michael et al. 1957). These studies identified factors such as spatial visualization, spatial relations (or speeded rotation), and spatial orientation. However, results differed, depending on the specific tests included in the battery and the factor-analytic technique used. More recent proposals for the structure of spatial skills include models with as few as one factor (Malanchini et al. 2020) and as many as 25 (Buckley et al. 2018).

### 3.2. Frameworks Lack Clarity

Frameworks that have been proposed often lack precision, leaving them open to interpretation. For example, Newcombe and Shipley (2015) proposed that spatial thinking can be classified according to two dimensions: intrinsic versus extrinsic and static versus dynamic. Static versus dynamic refers to the degree to which the object or information is moved or transformed; a good example is mental rotation. Intrinsic versus extrinsic refers to whether the representations encode spatial properties intrinsic to an object (e.g., shape, part–whole relationships) or relative to an external object or reference frame (e.g., configurations of landmarks). Although the extrinsic skills are sometimes conceptualized as primarily aligning with navigation (Newcombe 2018), they are sometimes interpreted as including small-scale, object-based tasks like the water level task and rod-and-frame task (e.g., Uttal et al. 2013). Given the importance of the distinction between large-scale and small-scale spatial thinking skills (Montello 1993; Hegarty et al. 2006), it may be that we need to consider a two-by-three classification, dividing extrinsic into small- and large-scale.

### 3.3. Important Constructs Are Often Excluded

Perhaps most notably, some frameworks have paid little, if any, attention to navigation. Although a critically important aspect of spatial thinking and a focus of much research (including two lines of work that led to a Nobel Prize), navigation has usually been treated as something separate or distinct from other aspects of spatial thinking. The role of navigation in STEM success has been particularly neglected. Although most evidence for relations between spatial skills and STEM focuses on small-scale, object-centered skills, evidence is mounting for the relationship between navigation and STEM as well (e.g., Nazareth et al. 2019a). In part, the paucity of research on navigation may stem from the difficulty in assessing it. There are few tests of navigation, and many are either artificial (done using paper and pencil) or require very large spaces (and hence cannot be used across labs). More recently, the advent of accessible and inexpensive (or free) virtual reality navigation tests (e.g., SILCton, see Weisberg and Newcombe 2016, 2018) has made this disadvantage less of a concern. But, researchers are not likely to take advantage of these innovations unless more information is provided about how the measures can be accessed and how they differ from each other. A recent survey of VRs used with children (Nguyen et al. 2023) showed that only SILCton is available on an open-access platform (virtualsilcton.com, accessed on 2 January 2024). An additional test is available, but access to collected data requires contacting the authors of the system (van der Ham et al. 2020).

### 3.4. Frameworks Are Based on Old Tests, Methods, and Data

All theoretical frameworks are constrained by the data they seek to describe. In the case of spatial skills, there are many limitations in spatial tests used to generate those data, as described above. Many of the classic frameworks were developed from a limited set of tests used on samples that were not widely representative, using factor analysis techniques that are no longer considered cutting-edge. Despite advances in spatial research, even newly developed frameworks suffer from similar problems, like a selection of tests that may exclude important spatial skills.

## 4. Suggestions for Improvement

Having documented the problems with spatial tests and analyses of spatial thinking, we now consider possible solutions. In some cases, the proposed solutions are already in development, but, in other cases, the proposed solutions are more speculative. There are three areas of suggested improvements: (a) more and better data, analysis, and reporting; (b) better information about and access to tests; and (c) better, clearer, and more comprehensive theoretical frameworks.

### 4.1. Recommendation 1: More and Better Data, Analysis, and Reporting

#### 4.1.1. Use Larger and More Representative Samples

Most research on spatial testing and spatial thinking is conducted with small and non-representative samples. Too often, the sample is the traditional introductory psychology subject pool; the problems with this approach are now very well documented (e.g., Henrich et al. 2010). Moreover, typical samples are often small, and underpowered analysis can affect validation studies. For example, if the empirical basis for validation is that there is a significant correlation between an old and new test, low power is clearly an issue. Fortunately, there are several new approaches to these problems that have substantial potential to provide larger and more representative samples. Many of these new approaches involve the use of technologies that support internet-based data collection. Some examples include the following:

**Gamification**. The best-known example of a gamification approach for spatial research is Sea Hero Quest (Coutrot et al. 2018, 2022; Spiers et al. 2023). Participants play a navigation game on cell phones, and the data are shared with researchers. Participants advance through multiple levels, navigating a virtual boat through different oceans and seas. The tasks provide challenges that, like many video games (Gee 2003), are intrinsically motivating. People play the game extensively, sometimes for hours, without any benefit other than the sense of mastery that improves as they get more involved.

Sea Hero Quest was originally developed to provide early assessments of dementia symptoms, with the goal of identifying and perhaps treating problems before they become severe. Slight decrements in navigation skills have been found to predict later dementia. Although Sea Hero Quest still serves this purpose, it has taken off as a fun game as well. Consequently, it has allowed researchers to study navigation and spatial thinking, with more than three million players participating. The popularity and accessibility of Sea Hero Quest have allowed researchers to examine questions that might otherwise be intractable. For example, it has been indicated that average navigation ability is related to the GDP of a country (Coutrot et al. 2018). Although this possibility had been suggested before, no previous researchers could test it at anything that approaches the scale of Sea Hero Quest.

**“Synthetic Aperture” Testing.** The internet has also engendered models of testing that allow for free access to a variety of different tests, including spatial tests. One example is the International Cognitive Ability Resource (ICAR) (e.g., Revelle et al. 2020), which provides 11 tests of cognitive skills that are available for free to participants (and can be made available to researchers). ICAR takes advantage of its very large number of participants to shorten the length of the tests. No participant takes the same test. Instead, each participant takes a random sample of items that are calibrated to provide adaptation to the participant’s level of ability. Because of the large number of participants, correlation matrices between items can be established with sufficient precision (Synthetic Aperture Personality Assessment, SAPA, Revelle). Thus, ICAR and other developing sites provide a means for researchers to access many different cognitive tasks and compare the results to those of very large samples.

**Personal Insight Incentives.** There is much to learn from the recruitment methods that bring participants to the ICAR sites. ICAR neither pays participants nor offers them a game to play. Instead, it offers participants information about their own personality or intellectual abilities. Other sites that use such a model include The Music Lab (n.d.) (www.themusiclab.org, accessed on 2 January 2024), a website that uses citizen science to learn about how people create and perceive music. The site hosts several tests or games that participants can find and take. Tests are usually just a few minutes, and participants receive a report of their performance relative to the average. Their most popular test is their longest: “Test Your Musical IQ”, which takes around 20 min to complete but has collected data from over 2 million participants (though the site allows participants to perform the tasks multiple times). Similarly, Project Implicit (n.d.) (implicit.harvard.edu, accessed on 2 January 2024), a non-profit collaborative interested in implicit attitudes and stereotypes, hosts a site with many implicit associate tests (IATs) for dimensions including race, gender, sexuality, age, and more. Participants receive reports about the degree of their implicit bias on a particular dimension. These projects clearly demonstrate that a very large number of participants can be recruited without financial incentives if people have the opportunity to learn more about themselves.

**Online Virtual Environments.** Another technological advance is the use of virtual reality to create environments that support simulated navigation and assessments of this navigation. Although virtual reality has been used in spatial thinking research for decades (e.g., Loomis et al. 1999; Rothman and Warren 2006), the innovation is to provide standardized, readily available, web-based virtual environments that can be used to measure individual differences in navigation skills or modified for new experiments. One example is Virtual SILCton, which is a virtual environment based on the Amber campus of Temple University. Weisberg and Newcombe (2016) used Virtual SILCton to examine both qualitative and quantitative individual differences in navigation skills and, specifically, the ability to develop knowledge of the layout of a space from the experience of taking routes through the environment. Virtual SILCton is now freely available through the Open Science Framework and is giving researchers an opportunity to study navigation in the same or similar environments across many people.

#### 4.1.2. Provide Better Analysis and Reporting

Larger, more representative data sets will also enable analyses to improve theory and assessment. There are many spatial skills tests that purportedly measure the same skills, such as “mental rotation”, “perspective taking”, or “cross-sectioning”. However, these tests vary on features, such as the type of stimuli to be transformed, whether they are timed, the response mode, etc. Existing factor analytic studies of spatial skills are out of date and have not included many of the tests that are currently in use. Moreover, modern techniques such as confirmatory factor analyses can be used to test theories about subcomponents of spatial thinking, but need large, representative, data sets, which are currently lacking.

There is also a clear need for more psychometric analyses of existing tests. Tests either lack reliability and validity information or the psychometric analyses are outdated. Modern psychometric techniques such as item response theory have not been applied to most spatial thinking measures. These techniques can reveal which items on a test are most diagnostic and how many items of each task are needed for efficient and reliable measurement. Researchers often adapt existing tests, such as by shortening them, adapting them for group administration, or developing online versions, without reporting the basic psychometric properties of these adapted tests.

Beyond psychometrics, best practices for data analysis and interpretation are not always followed. Researchers often overinterpret results (e.g., small differences in the strength of correlations between spatial skills and STEM outcomes), without reporting whether these differences were statistically significant. Moreover, they often do not correct for the reliability of tests when interpreting correlations. For example, researchers may conclude that there is a dissociation between the skills measured by two tasks based on a low correlation, when in fact that low correlation is due to a lack of reliability of the individual measures (Ackerman and Hambrick 2020; Hedge et al. 2018; Parsons et al. 2019).

Based on these issues, we recommend funding the collection of large, representative data sets that allow us to establish current norms for spatial thinking measures and enable us to apply modern techniques including item response theory and confirmatory factor analysis. We also recommend the development of minimal standards for reporting individual differences in any publication (like current standards in many journals for reporting power analyses and effect sizes). These should include reporting the reliability of each test in the current administration and evidence for validity, in addition to properties of the distribution (mean, standard deviation, skewness, and kurtosis). Other standards might include reporting tests of significance for differences between correlations and computing disattenuated correlations (Parsons et al. 2019; Spearman 1904) that take the reliability of the measures into account. We also note that these recommendations will require some education of the research community as many users of spatial thinking measures have not had formal training in psychometrics and are probably unaware of the relevant issues.

### 4.2. Recommendation 2: Seek Better Information on and Access to Spatial Tests

As reviewed in previous sections, the lack of information about spatial tests is a barrier for researchers. Tests often lack information about reliability and validity. In addition, researchers often do not know which tests to use in each context, or even exactly which tests measure which constructs. The researchers we interviewed were enthusiastic about solutions that would increase access to spatial tests and associated information, including the following:

#### 4.2.1. Create a Catalog of Spatial Tests

An updated, comprehensive catalog of available tests would support researchers in identifying and evaluating appropriate tests. The Buros Center for Testing (buros.org, accessed on 2 January 2024) provides a compendium of psychological tests in their Mental Measurements Yearbook series, which is an encouraging model for maintaining a catalog of tests. However, a look at their comprehensive index of reviewed tests reveals almost no spatial tests. At the time of writing, the list included only five tests with “spatial” in the name (out of 4041): (Brief Visuospatial Memory Test—Revised, Minnesota Spatial Relations Test—Revised, Spatial Awareness Skills Program Test, Spatial Orientation Memory Test, and Spatial Reasoning). There were no tests whose names contained terms for the spatial skills “mental rotation” (the most used type of spatial test as reported in our structured interviews), “navigation”, “paper folding”, “perspective taking”, or “cross-sectioning”. This absence is of course not due to a lack of such spatial tests—it stands in stark contrast to Eliot and Smith’s (1983) *An International Directory of Spatial Tests*, a published volume including the names and information of 392 tests of spatial skills, including test instructions, practice or sample items, scoring instructions, and availability. Unfortunately, this resource is now several decades out of date. More recently, Northwestern University’s SILC Resources page (https://www.silc.northwestern.edu/tests-and-software/, accessed on 2 January 2024) compiled a list of spatial tests, tools, and software. However, this resource is not an inclusive list, and it is challenging to keep it current, let alone expand it.

A new, online catalog of spatial tests would need to be comprehensive, accessible, and flexible enough to keep pace with a dynamic field. To remain useful, it would need to be updated and maintained long-term. The catalog should include (at least) the following: the spatial skills each test is intended to measure, the age groups and populations it is appropriate for, the duration of administration, any requirements for administration (e.g., special equipment), whether there are different versions of the test, where to find the test, who holds the copyright, the cost of the test, whether there are validity and norming data available, and a sample of empirical reports using the test.

The spatial test catalog would not only help researchers identify appropriate tests but would also help unearth shortcomings in existing tests, such as constructs that have no or few assessments (e.g., navigation), tests that require more validity/reliability data, or areas where we lack measures for certain age groups. That is, it would serve not only as a resource but also as a guide for future research and development.

#### 4.2.2. Make Tests and Documentation Accessible

Information about tests is vital, but researchers also need to be able to locate and acquire tests, along with any administration and scoring instructions or other associated documentation needed to administer the test correctly. It is easier now than ever, in the age of widespread internet access and cloud storage, to make things widely accessible. The open science movement has converged on guiding principles and practices—and, in some cases, new infrastructure (e.g., OSF—the Open Science Framework)—that likewise make sharing information and resources more manageable than ever before. There are several avenues by which test accessibility could be improved:

**Existing Infrastructure.** Individual researchers could make their tests and materials available on sharing sites like OSF. This option requires little additional investment toward sharing tests specifically and it capitalizes on the momentum of the open science movement, whose principles are increasingly being adopted as part of researchers’ standard practice. Field-specific guidelines for sharing tests—including things like the kind of information and materials to include, recommended formats and platforms, and other guiding principles—could improve the quality of sharing. There are many models to follow to develop these guidelines, with standards for different disciplines and methodologies already laid out in many cases.

However, this option leaves the onus of sharing, updating, and maintaining resources to individual researchers. Aside from this and other calls for researchers to share test information, there may be little to inspire field-wide change in sharing practices—those who are already able and willing to share may already be doing so through existing channels. In addition, by itself, this option makes minimal improvements in helping researchers identify relevant spatial tests—researchers must already know what they are looking for and where to find it. This approach would be most effective in conjunction with a comprehensive catalog of spatial tests detailing where these tests could be found.

**Centralized Repository of Tests.** A centrally managed test repository would address the cataloging and access issues together. One model for this is the existing SILC resources page, with tests and documentation available for download (or linked to other hosting sites, when appropriate), but scaled up to be more comprehensive and robust. Ideally, the resources provided in a repository would be free and open-source and would not rely on expensive software to administer, whenever possible. The repository could be fully maintained by a single group or, as some of our focus groups suggested, it could be a Wikipedia-style resource with standards and conventions for accessing and contributing to the database (including qualifications and appropriate credit for researchers who contributed). Ideally, it would also include infrastructure that enabled users to interpret their results, including citations to previous research that used the test. Another suggestion was to incorporate an “Ask an Expert” forum or “FAQ” section to enable researchers to obtain input and guidance for issues outside their areas of expertise. However, informants also noted the tradeoff between making tests available to researchers and not making them so available that people could “teach to the tests”.

**Testing Platform.** Perhaps the most ambitious option is to build a repository within a testing platform, from which researchers could directly administer the tests. Not only would this increase access to spatial tests but it would also ensure standardized administration and scoring, which was a common concern among the researchers we interviewed. There are several models for how this might be achieved, some of which are discussed in previous sections. Some are directly consumer-facing, with any individual able to participate (e.g., the first iteration of Sea Hero Quest). These have great potential to collect large data samples, but they are not generally open to those beyond the research team to access the tests themselves.

The models with the most potential to serve the wider research community are those that make tests available for use by other researchers, with some ability to tailor administration for their project needs. Examples include the new iteration of Sea Hero Quest, SILCton, and ICAR. ICAR offers a battery of tests for different skills and allows researchers to customize what they administer. Something like ICAR specifically for spatial tests could allow researchers to easily identify, administer, and score validated and standardized tests for a variety of spatial skills.

It is likely that a testing platform would need to offer a smaller, curated battery of tests compared to a comprehensive repository. Thus, this option might be best implemented once a better foundation has been built by surveying and gathering psychometric information about existing tests.

#### 4.2.3. Challenges

There was enthusiasm from our respondents about resources to improve access to tests. However, even the simplest option—compiling a comprehensive catalog of spatial tests—will require a significant investment of time to review the literature and reach out to researchers, especially at the outset. This effort will require some expertise in spatial assessment and research. Technical expertise may also be needed to build the database itself. Keeping the content of the database current is also an ongoing investment—someone must continue to stay abreast of new developments including new tests, new versions of old tests, and new information about tests, and this effort must continue as long as the resource is available. In addition, continuing technical and user maintenance (fixing bugs, answering email inquiries, etc.) is necessary to ensure utility. In addition, many researchers may also be wary of making their tests available without conditions or assurances of their use due to concerns about test security (e.g., “game”ing the tests, becoming too familiar with the tests) or unqualified users misusing the test.

Compared to a catalog of tests, building a repository of tests is a much larger undertaking. It will require an organized effort and contributions from the community to be executed. Individual researchers may be reluctant to contribute their tests to a centralized repository without some incentives like making tests and psychometric reports citable. In addition, many tests may have copyright restrictions that disallow open sharing. Some tests could be designed to use an algorithm like ICAR’s (so that no participants see the same set of items) to guard test security. Access to the tests could also be limited to qualified researchers through, for example, an application or registration process.

However, these considerations highlight the overarching challenge: who will lead the creation and maintenance of these resources? And of critical importance is the question of how they will be funded.

### 4.3. How Do We Create the Tools We Need?

To develop an organizing theory of spatial thinking, we need more and better data (and, in turn, better theory will drive new, fruitful empirical investigations). More and better data will require increased information about and access to spatial tests. Thus, progress in the field depends on the mutual relationships between theoretical insights, better data, and better access to spatial tests. Although there are some actions we can take now to improve the state of spatial research—for example, creating guidelines for data analysis and reporting—tools to enable large-scale data collection and make tests more accessible are potentially massive undertakings. Moreover, we must ensure that any solutions can be maintained and adapted over time to continue to support researchers’ needs.

#### 4.3.1. Engaging STEM Experts

Critically, the development of assessment tools and infrastructure must engage STEM experts. Although we know that there is a correlation between spatial skills and achievement in STEM, and there has been substantial progress in understanding this relation in the domain of mathematics (e.g., Gunderson et al. 2012; Hawes and Ansari 2020; Mix 2019), progress is slower in other STEM domains. Because researchers in various STEM disciplines do not have a comprehensive theory as to how spatial thinking is used in their field, the choice of tests is often hit-or-miss, guided by historical conventions or word-of-mouth, with little knowledge or consideration of which test would be best and why.

Conversations with STEM experts are needed not only to determine which spatial tests are best suited for which disciplines but also to shed light on how and why spatial reasoning matters in their respective disciplines. Spatial reasoning in STEM is a complex process that relies both on knowledge of the material (e.g., possible structures of molecules or ways in which geological structures break or fold) and spatial skills, and the two may work together in unexpected but important ways (Atit et al. 2020). For example, Stieff (2007) found that chemistry experts did not need to mentally rotate symmetric molecules to make decisions about chirality (i.e., whether two molecules had the same structures or were mirror images); they used analytic strategies that could also be taught effectively to students, regardless of their spatial abilities. Consultation with STEM experts can shed light on spatial processes that had not been considered before. A good example is Atit et al.’s (2013) work on non-rigid transformations and their importance in geometry (discussed above); see Atit et al. (2020) for additional examples.

#### 4.3.2. Maintaining a Test Catalog and Testing Platforms

The technical requirements of these proposals are quite low. For example, the cost of servers and storage is now negligible. The biggest expense instead is for personnel. Building and maintaining these resources will require some expertise in areas like spatial research and testing, psychometrics, database and web development, and project management. In many ways, such a project is not suited to be assigned solely to graduate students or postdocs, who often carry out much of the day-to-day work of research. Many aspects of creating these resources will not involve research questions that would be appropriate for developing a research program or dissertation (in spatial thinking or STEM). Rather, dedicated staff with the requisite skill sets and more permanent appointments are needed.

**Models from Existing Platforms.** Here, we describe in some detail two existing online platforms with spatial tests, SILCton and ICAR. We believe these are the “bleeding edge” of online, sharable testing systems. We outline the requirements and challenges of each, but particularly highlight the possibilities for those mentioned above. These platforms and new ones that they inspire can be expensive, but the benefits may well be worth the cost. Moreover, as their importance and value become clearer, more organizations or individuals may contribute to the development.

***SILCton*.** SILCton was created over a decade ago at Temple University and is currently being maintained by Steven Weisberg at the University of Florida. SILCton is a web-based virtual reality (VR) platform to test navigation. The VR environments are built in UNITY and are connected to a database and login system, with different levels of access to the data. Researchers request access from Dr. Weisberg, who performs a basic check to make sure requests are legitimate (e.g., the requester has a reasonable justification for wanting access, has a verifiable web presence, can provide a CV, etc.), and researchers must register on the site to gain access. The platform is hosted on an Amazon server. Participants who are directed to SILCton participate in a single study, which is nested within a “lab”. Each study customizes its instructions, measures, and orders. Labs must maintain their own IRB approvals for their studies, but the ability to collect data and store it on an anonymized database is covered by an IRB managed at Temple University.

For now, SILCton is being maintained as is, with no significant changes, improvements, or upgrades planned. The bare minimum to keep it operating and available still requires a fair amount of time. A web developer, kept on retainer for a certain number of hours each year, is essential to keep SILCton running in case anything goes wrong or the infrastructure needs to be dramatically updated or changed. The web developer also fixes bugs and makes small changes as needed. Weisberg manages the project and communicates with the web developer, in addition to answering emails from researchers, updating documentation, performing technical maintenance, creating and maintaining the suite of tools researchers need to use SILCton (e.g., analysis code), and dealing with any issues that arise. Among these, only a few tasks could be outsourced to a less-expert staff member (answering emails, updating documentation).

In some ways, SILCton is a fairly simple test platform model to execute. It offers just one type of assessment for one type of spatial thinking (navigation). In addition, SILCton is merely being maintained, not improved. To be clear, this is not because SILCton already achieves all it is desired to. As Dr. Weisberg stated plainly, “If I had more to spare, there’s plenty I’d build into it”. But expanding functionality—for instance, to enable a citizen science approach and make it available directly to the public—entails a host of additional demands and potential issues that would require a considerably larger investment than it already has.

***ICAR*.** The International Cognitive Ability Resource (www.icar-project.org, accessed on 2 January 2024) is a web-based, public-domain assessment tool made up of a battery of validated tests of cognition (currently, there are 11). ICAR has been developed over the last decade. It was initially managed by David Condon (who began the work as a graduate student at Northwestern University and is now at the University of Oregon) and is now primarily managed by collaborators in Germany. Some of the tests are inspired by automatic item generation: algorithms are created to generate items that can create a large repository of material that can be sampled for various goals (e.g., items that measure a specific feature, more or less difficult items, etc.).

The tests were initially validated through SAPA (Synthetic Aperture Personality Assessment, www.sapa-project.org, accessed on 2 January 2024) and, over the past several years, ICAR has introduced and validated additional measures of cognitive ability. Creating new tests and items involves searching the literature to see how a particular skill has been previously measured and reverse engineering the task to create an algorithm to generate a factorial combination of items. Although this seems relatively straightforward, it requires some expertise in the relevant cognitive skills and their assessment. Adapting the tests for online administration can sometimes be technically challenging. Validating the measures is also challenging since it requires multiple iterations of testing and therefore a large amount of data. Someone with expertise in psychometric methods is needed to perform the analyses.

Most challenging, however, is keeping the project staffed and funded. In addition to finding, designing, and validating the measures—which requires some combination of content area expertise, technical (e.g., coding) knowledge, and experience in psychometrics—other critical tasks include keeping resources and the website up to date, responding to emails, and granting and monitoring access. Many of these tasks are not a good fit for graduate students or post-docs, who have other priorities in their career path that typically involve developing and testing theories, for example. Thus, maintaining or growing the project requires paying for research, programming, psychometrics, and admin staff, but there is much less funding available to sustain existing research resources like ICAR than there is to support hypothesis-driven projects.

#### 4.3.3. Sustainability and Funding Models

As promising as these new models for test access and administration may be, they all currently may suffer from the issue of sustainability. Even if initial funding could be procured to create these tools (e.g., through traditional grants), maintaining and updating them will also require funding, and traditional grants may not be appropriate for the long term. In general, resources like these, which benefit the whole field, and which rely on at least some cooperation from the community, may be best served by a collaborative model that makes use of economies of scale and sharing resources. Here we lay out some existing models for collaborative and other types of funding:

**Collective Institutional Funding.** PsyArxiv uses this model. PsyArxiv is a widely used repository for preprints in the social sciences. It is funded through an institute collective, with all participating institutions contributing a small portion of the funding and providing input on the project. Because each institution invests only a small amount of funds, it does not place a large financial responsibility on any one party. Collectively, there is enough funding to sustain the resource long-term.

**Open Access.** Another way of distributing the cost of the resource is through an open-access model like those used in open-access publishing. In this case, contributors to the repository (i.e., researchers who provide tests and materials) would pay to have their test “published” using grants or institutional funds. In return, they would be provided with a citable DOI for anyone using the test. One drawback of this approach is that this would exclude from the repository any tests from researchers who are not willing or able to pay.

**Specialized Grants.** There are opportunities for specialized grants that are suited to building and maintaining resources for researchers. For example, NSF has a Research Infrastructure Program, which “provides support for the acquisition, enhancement, and operation of forefront computing research infrastructure and experimental facilities” (https://beta.nsf.gov/funding/opportunities/research-infrastructure-program, accessed on 2 January 2024).

**Tiered Access.** Another funding model that could provide long-term support is a tiered-access model. In this model, access to some features or parts of a resource is free or at a very low cost, and gaining access to additional features costs more. For example, access to a catalog of tests might be provided for free, but there would be a fee to gain access to the tests themselves. Tiers are flexible, so there might be a tier that allows downloading one test per month and another, more expensive tier that allows unlimited access and downloading. Whatever the tier structure, the goal is that the higher fees cover the maintenance of high-cost features and make maintaining the low-cost features efficient enough to be essentially cost-free.

**For- and Non-Profit Partners.** The preceding models could all be achieved by individual or collaborative groups. In contrast, this model involves forging a partnership (or sponsorship) with a for-profit company that may have an interest in spatial testing, such as Pearson, or a non-profit entity like the Educational Testing Service (ETS). These organizations have comparatively vast resources for developing and maintaining products. Other candidates are companies like Duolingo, which is interested in developing assessments in service of improving learning and has recently branched out beyond language learning to mathematics.

### 4.4. Recommendation 3: Develop a Coherent Theory of Spatial Skills and Spatial Assessments

It is critically important to develop a coherent theory of spatial skills and assessments. As an example of the difference a coherent theory can make, consider the progress that has been made in understanding the nature of personality and its assessment. Although there is certainly still disagreement about what personality is, how it should be measured, etc., much of these debates have converged towards the “Big Five” theory, which specifies five dimensions of personality (openness, conscientiousness, extraversion, agreeableness, and neuroticism). The development and assessment of the Big Five have made personality psychology a more coherent and visible sub-field, and measures developed based on the Big Five can specify both what they measure, how well, and whether new knowledge has been added (e.g., Revelle 1995). Even if one disagrees with the Big Five, it nevertheless provides a framework for disagreement, discussion, and progress toward consensus.

Although a coherent theoretical framework is critical, it depends on adequate information about spatial skills and how they relate to one another. As we have reviewed above, we do not have this knowledge, and so we must first solve issues with testing and data to gain purchase on developing any kind of useful framework. The proposed resources could lead to the iterative building and testing of conceptual models that would have the potential to become clear and persuasive.

## 5. Moving Forward

Our long-term vision for the future has a robust infrastructure for supporting research on spatial thinking—one in which researchers and educators have ready access to information and tools to make spatial testing easier, more accurate, and possibly less expensive, which, in turn, will support coordinated, efficient efforts to explore and support spatial thinking. Such an infrastructure would be invaluable, but it is also quite ambitious. As a first step, we call for a systematic review of spatial tests and theories of how spatial skills relate to one another. The review would provide a thorough overview of the current state of spatial tests and theories, reveal areas for improvement, and highlight important questions. Producing a systematic review will require compiling a comprehensive table of information about spatial tests, how they have been used, what constructs they are intended to measure, correlations with other spatial measures, psychometric information when available, and other information. This table would be of immediate value to the research community on its own (shared openly, e.g., on OSF). It also would provide the information to seed a dynamic, user-friendly catalog of spatial tests that could be updated and maintained over the long term to continue to serve researchers and educators.

The infrastructure we have proposed would help bridge the research–practice gap by providing access to information and assessments that will support STEM educators and practitioners in exploring and supporting spatial thinking. Infrastructure for assessing spatial skills will accelerate and improve research efforts, but it will also provide tools, like formative assessments, for STEM training and recruitment. It can also help to catalyze collaboration across fields, networks, and locations by providing a common resource accessible to all parties. A robust infrastructure for spatial thinking would position the U.S. as a global leader in an area drawing increasing interest as a key piece of STEM success. Thus, there is significant potential for the continued exploration and support of spatial thinking to advance our STEM workforce—if we can solve the practical and conceptual limitations outlined above.

Moreover, our proposed solutions would help to unify the field and accelerate progress and insights. For example, STEM education researchers seeking to understand how spatial skills relate to success in their discipline would have a resource to make principled decisions about what spatial constructs would be of interest and which spatial tests would be most appropriate to examine them, rather than being limited by whichever spatial tests they can access. Open sharing of data and materials would enable data-driven insights that are out of reach with the current state of affairs. A robust infrastructure for spatial thinking research, making use of modern innovations in technology and testing, would serve as a model for other fields.

## 6. Conclusions

With the increase in spatial thinking research in the last few decades, we are now realizing the benefits of NSF and other funders’ investment in research on spatial thinking (Stieff et al. 2014). In many STEM fields, but particularly in mathematics, we now have a much clearer understanding of when, why, and how spatial thinking matters for learning, achievement, and persistence (e.g., Atit et al. 2020; Mix 2019; Hawes and Ansari 2020). We are now witnessing the development of several evidence-based programs to facilitate spatial skills as they relate to STEM learning (e.g., Hawes et al. 2022; Larkin and Lowrie 2022; Lowrie et al. 2021). As important as these efforts and outcomes are, progress is greatly hampered by the limitations in spatial testing and theory that we have documented here. However, the news is not all bad; we have made several actionable suggestions for improvement. If implemented, these improvements would greatly reduce the piecemeal approach to understanding spatial thinking that has characterized the field thus far. Our hope is that this article will launch a major new effort to understand what spatial tests measure, make tests more accessible and easier to use, and promote the growing network of researchers who study how spatial thinking is related to STEM.

## Data Availability

Not applicable.

## Appendix A

### Introduction

The goal of this survey is to collect information about the use of spatial tests in research. The results of this survey will be used to identify which spatial tests are used by the research community, how they are used, and what benefits or challenges they pose. We are interested in all kinds of spatial tests that use objective performance measures (i.e., not self-report only), including small-scale tests and tests of navigation (including VR environments); standardized and non-standardized tests; and so on.

This survey is part of a larger project aimed at developing an interdisciplinary, evidence-based consensus regarding what different spatial tests measure, how they are related to each other, and where there are gaps in existing spatial tests. This information is critical for the field to continue moving forward productively.

Thank you for contributing your time and expertise to our survey.

Q1: Please list the **top 1–3** spatial tests that you use in your research. You will be asked a few **follow up questions** about these tests later.
Please list any other spatial tests you use in rows 4–10. You will not be asked any more questions about these tests.

	List the **top 1–3** spatial tests that you use in your research. List any additional spatial tests in rows 4–10.			The test has been:			Is there a manual or detailed instructions for administration and scoring available?			
	Test Name (1)	Spatial skill(s)/ability(s) tested (2)	Age range test is appropriate for (e.g., 4–12 years) (3)	Validated (1)	Normed (2)	I’m not sure (3)	No (1)	Yes, from the researchers (2)	Yes, publicly/commercially available (3)	I’m not sure (4)
* **1***				□	□	□	□	□	□	□

The following questions are about **[Test 1*]**. (* repeated for each of the top 1–3 tests listed).

Q2: What is the format of [Test 1]? How is it administered? (select all that apply)
  □ Individual administration (1)
  □ Group administration (2)
  □ Paper/pencil (3)
  □ Computer-based (4)
  □ Online/web-based (5)
  □ Requires special equipment/setup (e.g., eye-tracker, VR, etc.). Please describe:
    (6) __________________________________________________
  □ Other (please describe:) (7) __________________________________________________
Q3: How did you get access to [Test 1]? (select all that apply)
  □ I am/was involved in its development (6)
  □ I bought it (1)
  □ I found it online (2)
  □ I received it from the test developers or copyright owner and am using it with their restrictions (3)
  □ I received it from another researcher (who is not the developer or copyright owner) (7)
  □ I had a paper version and made my own online version (5)
  □ Other (please describe:) (4) __________________________________________________
Q4: How do you use [Test 1] in your research? (select all that apply)
  □ As a measure of general spatial skill (4)
  □ As a measure of specific spatial skills (5)
  □ To measure individual differences (6)
  □ To measure group differences (7)
  □ To examine spatial skill over development (i.e., with age or experience) (8)
  □ To examine relationships between spatial skill(s) and other areas of performance (9)
  □ To understand relationships between different spatial skills (10)
  □ As a reference to develop other spatial tests (11)
  □ To evaluate the properties of the test (e.g., its validity) (12)
  □ Other (please describe:) (13) __________________________________________________
Q5: What are the advantages of [Test 1]? (select all that apply):
  □ Availability of test (4)
  □ Availability of information about the test (5)
  □ Validity or reliability (6)
  □ Ages or populations the test can be used with (7)
  □ Frequency of use by others (8)
  □ Cost (9)
  □ Ease of administration (10)
  □ Mode of administration (11)
  □ Duration of test (12)
  □ Flexibility or adaptability (13)
  □ Overlap with other tests (14)
  □ Other (please describe:) (15) __________________________________________________
Q6: What are the disadvantages of [Test 1]? (select all that apply)
  □ Availability of test (4)
  □ Availability of information about the test (5)
  □ Validity or reliability (6)
  □ Ages or populations the test can be used with (7)
  □ Frequency of use by others (8)
  □ Cost (9)
  □ Ease of administration (10)
  □ Mode of administration (11)
  □ Duration of test (12)
  □ Flexibility or adaptability (13)
  □ Overlap with other tests (14)
  □ Other (please describe:) (15) __________________________________________________
Q7: Are there any caveats about [Test 1] you think are important to share?
  ________________________________________________________________

## Appendix B

The purpose of this interview/focus group is to gather information about the needs of the spatial cognition and education communities regarding tests of spatial abilities and skills. Through this interview we hope to understand how you use spatial tests now, any theories (including taxonomies) that guide your research, and what spatial tests and infrastructure for spatial testing you would like to see to support your research.

**Questions about interviewee:**
  1. What is your current position (e.g., professor, grad student, postdoc) and what is your expertise (e.g., engineering, education, psychologist)?
**Questions about spatial tests:**
  2. Why are you interested in Spatial Abilities testing (e.g., want to understand the capabilities of your students, measure their learning, basic research)?
  3. Are there specific spatial skills you’re interested in measuring?
  4. What test(s) do you currently use?
  5. How/where do you find information about tests?
  6. How do you decide which tests to use? Has it ever been a challenge to your research to locate (identify, find) the tests that you need?
  7. What would an ideal spatial measure be for you? What would it measure? How would it be administered? To whom and in what contexts would it be used? What would you do with it?
**Questions about taxonomies/theories:**
  8. Do you have/use any particular theory of spatial skills to guide your research? (e.g., taxonomies based on factor analysis, the 2 × 2 taxonomy developed by Newcombe & Shipley (intrinsic/extrinsic & static/dynamic))?
  9. What do you think such a theory needs? What should it be able to do, how could it be used, what needs to be answered for it, or by it?
  10. Is such a theory helpful or necessary for your research?
**Broadly what do you need from Cognitive Psychologists:**
  11. We have been considering trying to seek funding to build resources for the field. Examples of what we propose to do are standardizing existing tests, making tests available via open source repositories, and making fully online versions of tests.
  12. Would these resources be of interest to you, or can you think of other types of infrastructure that we might develop?
  13. Do you have any other suggestions for how cognitive psychologists could support your research?
